# Electrical coupling between transplanted cardiomyocytes and host myocardium to prevent arrhythmia

**DOI:** 10.1242/dmm.052768

**Published:** 2026-07-03

**Authors:** Bijay Guragain, Lei Ye, Jack M. Rogers, Jianyi (Jay) Zhang

**Affiliations:** ^1^ Department of Biomedical Engineering, School of Medicine and School of Engineering, University of Alabama at Birmingham, Birmingham, AL 35294, USA; ^2^ Department of Medicine/Cardiovascular Diseases, University of Alabama at Birmingham, Birmingham, AL 35294, USA

**Keywords:** hiPSCs, Graft-host coupling, Graft-associated arrhythmias, Myocardial infarction, Cell therapy

## Abstract

Transplantation of human induced pluripotent stem cell (hiPSC)-derived cardiomyocytes offers new opportunities for myocardial repair after infarction. However, as demonstrated in large-animal model studies, such therapy also brings translational challenges, including arrhythmias arising from abnormal spontaneous beating of the engrafted cells or irregular conduction due to poor electrical coupling between host and transplanted tissue. Addressing these issues will have important implications for improving the safety and efficacy of regenerative therapies. This Review summarizes the fundamental mechanisms governing cardiac electrical activity and highlights recent technological advancements for triggering and imaging myocardial electrical function. We focus on emerging experimental platforms that overcome limitations of traditional whole-heart mapping approaches, including organotypic myocardial tissue slices combined with high-resolution optical mapping and optogenetic stimulation. We further discuss recent technological and biological developments in the field of cell transplantation for cardiac repair and examine strategies to manage post-transplant arrhythmia risk, with a particular focus on enhancing graft maturation and electrical integration to accelerate the safe and effective clinical translation of cardiac cell therapies. Finally, we describe recent clinical trials involving transplantation of hiPSC-derived cells into damaged hearts.

## Teaser

**Summary:** This Review examines recent developments in cardiomyocyte-based therapies for heart disease, with a particular focus on electrical coupling between graft and host tissue and the risk of graft-associated arrhythmias.

## Introduction

Cardiovascular disease (CVD) is the leading cause of death worldwide and affects individuals across all racial and ethnic groups. In the USA, nearly half of all adults (48.6%) have CVD, equating to a total economic burden estimated at nearly $1 billion per day (National Center for Health Statistics). More than 40% of CVD diagnoses are for coronary artery disease (Martin et al., 2024), which often leads to postinfarction left-ventricular remodeling. Although the mortality rate for myocardial infarction (MI) has declined from 1 in 2 in the 1950s to 1 in 10 now (Palaniappan et al., 2026), surviving patients with large infarcts frequently experience a progressive decline in cardiac performance that eventually leads to heart failure (Jenca et al., 2021).

Up to 1 billion cardiomyocytes can be lost during a single MI event (Hashimoto et al., 2018; Kikuchi and Poss, 2012; Laflamme and Murry, 2011; Zhao et al., 2020). However, only 45% of cardiomyocytes are replaced via self-replication over a person's lifetime (Bergmann et al., 2009); thus, the intrinsic proliferative capacity of adult human cardiomyocytes is far too low to regenerate those lost cells due to an MI. In addition, post-MI arrhythmias, including ventricular tachycardia and fibrillation, remain a major cause of morbidity and sudden cardiac death. Current therapies such as implantable cardioverter-defibrillators and cardiac resynchronization therapy can reduce mortality and improve ventricular synchrony, but they do not repair damaged myocardium (Zhang et al., 2024). The only currently available treatment option that can fully restore myocardial integrity and performance in patients with heart failure is whole-heart transplantation, which is constrained by the limited availability of donor hearts (Brown et al., 2024). Consequently, researchers continue to investigate novel methods for repopulating the scarred regions of infarcted hearts with functioning cardiomyocytes.

Strategies for implanting exogenous cardiomyocytes or engineered cardiac tissues have been studied for more than three decades (Sadek and Olson, 2020; Ye et al., 2014; Zhao et al., 2021; Zhu et al., 2018). Transplantation of these cells or tissues has demonstrated remuscularization of damaged myocardium with promising therapeutic effects, including improved cardiac function, reduced scar size and reduced ventricular wall stress (Ye et al., 2014). However, arrhythmias associated with the transplanted tissue remain a major safety concern for clinical translation. The experimental model used to evaluate transplantation is particularly important in this regard: transplantation-associated arrhythmias are not typically observed in small-animal models (mice, rats and guinea pigs) (Shiba et al., 2012), likely owing to their smaller heart size and higher heart rate. However, they are frequently reported in clinically relevant large-animal models [pigs (Kawaguchi et al., 2021; Selvakumar et al., 2024) and nonhuman primates (Chong et al., 2014; Kobayashi et al., 2024; Liu et al., 2018)]. In addition to transplantation, there are recent and innovative approaches to treat infarcted heart that either activate proliferation of native cardiomyocytes via upregulation of cell-cycle regulatory molecules (Pasumarthi et al., 2005; Shapiro et al., 2014; Xiang et al., 2016) or genetically reprogram fibroblasts into cardiomyocytes (Fu and Srivastava, 2015; Tani et al., 2023; Yamakawa and Ieda, 2021; Zhao et al., 2015). Regardless of the remuscularization approach, the engrafted or regenerated cardiomyocytes must contract in concert with the native myocardium, so integration with the heart's endogenous electrophysiological system is crucial both to improve cardiac performance and to avoid arrhythmogenic complications. Thus, regenerative therapies are both a promising opportunity to restore coordinated cardiac function and a critical safety challenge, as incomplete or improper integration may exacerbate arrhythmogenic risk.

This Review begins with a discussion of the structures and mechanisms that govern cardiac electrophysiology, as well as recent advancements in technologies for monitoring the electromechanical properties of the myocardium. We then provide a brief summary of the progress made and challenges encountered during the development of cardiomyocyte-based therapies for heart disease, with a particular focus on how inadequate coupling between the implanted and native cells can contribute to the risk of arrhythmia.

## Cardiac electrophysiology

MI causes structural and electrophysiological remodeling that promotes arrhythmias by disrupting electrical function, altering ion channel function and creating fibrotic, heterogeneous tissue substrates (Donahue et al., 2024). These changes increase the risk of abnormal electrical conduction (Donahue et al., 2024). Understanding this disrupted electrophysiological environment is essential when implanting stem cell-derived cardiomyocytes or engineered tissues, as poor electrical integration can exacerbate graft-associated arrhythmias and hinder safe clinical translation (Chong et al., 2014; Nakamura et al., 2021; Shiba et al., 2016).

**Fig. 1.**
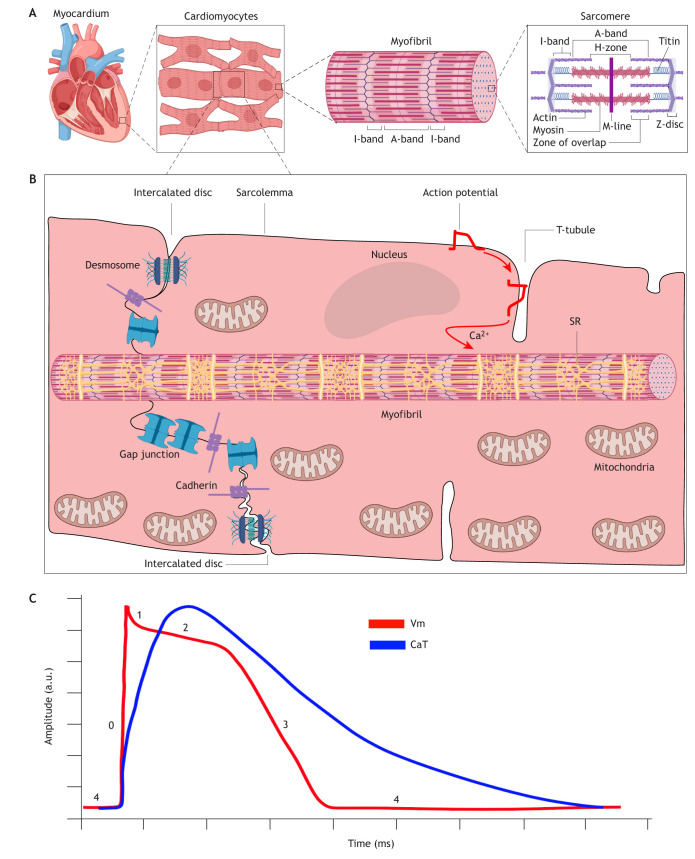
**Anatomy and physiology of myocardium.** (A) Composition of myocardium, showing cardiomyocytes containing contractile units (myofibrils) composed of repeating sarcomeres with actin and myosin filaments arranged into I-band, A-band, H-zone, M-line and Z-disc. (B) Cardiomyocyte internal structures and excitation-contraction coupling. Intercalated discs with desmosomes and gap junctions enable electrical and mechanical coupling. Membrane potentials propagate via T-tubules, trigerring Ca^2+^ release from sarcoplasmic reticulum (SR) to initiate contraction. (C) Cardiac membrane potential (Vm; red) with five phases from 0 to 4 (depolarization to repolarization) and corresponding calcium transient (CaT; blue). a.u., arbitrary units. Created in BioRender by Guragain, B. (2026). https://BioRender.com/hsyop48. This figure was sublicensed under CC-BY 4.0 terms.

Cardiomyocytes are striated and branched muscle cells that measure 10-20 μm in diameter and 50-120 μm in length (Karperien et al., 2019; Ripa et al., 2024). Mammalian cardiomyocytes usually contain one or two nuclei (Bishop et al., 2022), 5000-8000 mitochondria, and a sarcoplasmic reticulum that functions to store, release and reuptake calcium (Ca^2+^) ions essential for muscle contraction and relaxation (Fig. 1) (Ripa et al., 2024; Saxton et al., 2024). The contractile activity of a cardiomyocyte is performed by myofibrils, which consist of a repeating series of sarcomeres, each of which is composed of long proteins organized into thick (myosin) and thin (actin) myofilaments (Saxton et al., 2024) that drive contraction via the ‘sliding filament’ mechanism (Powers et al., 2021). Cardiomyocytes are connected to each other via intercalated discs that contain desmosomes, fascia adherens and gap junctions (Fig. 1B) (Rampazzo et al., 2014). Desmosomes and fascia adherens include cadherins and other adhesive proteins that form mechanical junctions between adjacent cells (Rampazzo et al., 2014), while gap junctions are composed of connexins that facilitate electrical coupling (Kanno and Saffitz, 2001; Ripa et al., 2024). These connexins have been categorized into 15 subtypes based on their molecular masses; connexins 30.2, 40, 43 and 45 (CX30.2, CX40, CX43 and CX45, respectively) are expressed in mammalian hearts, and CX43 is the most common (Paci et al., 2020; Yanamandala et al., 2017).

The heart's electrical conduction system controls its rhythmic contractions. There are a variety of electrically active cell types that are able to depolarize and fire electrical impulses known as action potentials (APs). For example, cells in the sinoatrial (SA) node depolarize spontaneously and beat rhythmically without external stimulation (Wei et al., 2025). This property is called automaticity, and these cells drive the normal heartbeat. Other cell types behave differently. For example, ventricular cardiomyocytes maintain a stable resting potential of approximately −90 mV and normally depolarize only when excited by electrical input from adjacent cells. Depolarizing currents spread through gap junctions, raising the membrane potential of the ventricular cardiomyocytes to approximately −70 mV and triggering the opening of fast voltage-gated sodium (Na^+^) ion channels. This initiates the first of the five distinct phases of the ventricular AP (Fig. 1C), which are summarized as follows (Grant, 2009):

- Phase 0 (rapid depolarization). Fast Na^+^ channels open, which allows the rapid influx of Na^+^ and sharply increases the membrane potential (Vm). Once Vm reaches −40 mV, L-type Ca^2+^ channels also open to allow the influx of Ca^2+^.
- Phase 1 (initial repolarization). Depolarization peaks around +20 to +30 mV, at which time the fast Na^+^ channels close, potassium (K^+^) channels open and the cell partially repolarizes as K^+^ ions exit the cell.
- Phase 2 (plateau). K^+^ efflux balances Ca^2+^ influx, and Vm remains relatively stable for ∼200 ms.
- Phase 3 (rapid repolarization). Ca^2+^ channels close, but K^+^ channels remain open; thus, repolarization accelerates.
- Phase 4 (resting potential). Once Vm returns to −90 mV, K^+^ channels close, and ion concentrations are restored via Na^+^-K^+^ pumps and Ca^2+^ transporters.

The APs in cardiomyocytes described above trigger contraction via a process called excitation-contraction coupling, which is driven by fluctuations in intracellular Ca^2+^ levels called Ca^2+^ transients (CaTs), as illustrated in Fig. 1C (Sun et al., 2024; van Meer et al., 2019). The mild increase in cytoplasmic Ca^2+^ due to L-type Ca^2+^ influx activates ryanodine receptors that release a flood of stored Ca^2+^ from the sarcoplasmic reticulum (i.e. Ca^2+^-induced Ca^2+^ release). The freed Ca^2+^ displaces tropomyosin from the actin filament to activate the cell's sliding filament machinery (Fig. 1B,C) (Powers et al., 2021). The force of contraction depends on intracellular Ca^2+^ concentration, and relaxation occurs when Ca^2+^ is either pumped back into the sarcoplasmic reticulum by sarcoendoplasmic reticulum calcium ATPase (SERCA; also known as ATP2A) or expelled from the cell by (primarily) the sodium-calcium exchanger (NCX; also known as TLX2) (Bers, 2002; Eisner et al., 2017).

In the heart as a whole, the normal electrical activation sequence begins with APs generated in the SA node. The activation wave spreads through the atrial myocardium through gap junctions, causing coordinated atrial depolarization and contraction. The impulse then reaches the atrioventricular (AV) node, where conduction slows, allowing ventricular filling. From the AV node, the AP travels rapidly through the His-Purkinje system to the ventricular myocardium, initiating ventricular contraction. This coordinated wave ensures synchronized atrial and ventricular contraction and efficient cardiac output. Disruptions to the normal activation sequence are known as arrhythmias (Grant, 2009).

Arrhythmias are usually divided into two broad types. Reentrant arrhythmias (Goyal et al., 2024) occur when an activation wave circulates in a loop and reactivates tissue it has already visited. One type of reentry is called anatomic, meaning that the wave circulates around an in-excitable central obstacle, such as a scar. Anatomic reentry usually requires a region of slow conduction within the circuit, and, for initiation, requires a conduction block in one direction around the circuit but not the other (unidirectional block). Functional reentry is the other major type of reentry. It occurs when a section of a wave is blocked, forcing the wave to recirculate around its own broken end(s). There is no central in-excitable obstacle. Functional reentry is favored by electrical heterogeneity, such as localized regions in the tissue with markedly prolonged AP duration, which create spatial dispersion of repolarization and facilitate reentrant circuit formation.

The second broad arrhythmia type involves abnormal wave initiation from a focal site. Such focal activation might result from abnormal automaticity in the SA node or from early or delayed afterdepolarizations (EADs or DADs, respectively). Afterdepolarizations are abnormal, premature fluctuations in cardiac AP that occur during or immediately after the repolarization phase (phase 2, 3 or 4) of the AP. These spontaneous oscillations are collectively referred to as triggered activity. Triggered activity develops from aberrations in the movement of ions (Almeida et al., 2015; Paci et al., 2020; Qin et al., 2003). EADs typically occur when the influx of Na^+^ or Ca^2+^ exceeds K^+^ outflux, leading to ectopic beats during phase 2 or 3 of the AP (Paci et al., 2020). DADs are usually caused by Ca^2+^ overload in the myocyte, which triggers spontaneous Ca^2+^ release and activates the NCX. As the NCX brings in three Na^+^ ions for each Ca^2+^ ion expelled, there is a net influx of positive ions that depolarizes the membrane and produces ectopic beats in phase 4 (Paci et al., 2020).

Graft-associated arrhythmias are abnormal heart rhythms that arise following cardiac cell transplantation owing to multiple interacting mechanisms associated with the transplanted graft. Key contributors include incomplete or heterogeneous electrical coupling between grafted and host myocardium, which creates regions of slow conduction and block (Guragain et al., 2025). In addition, differences in ion channel expression and AP properties between transplanted and host cells can promote spatial dispersion of repolarization. Structural remodeling, including fibrosis at graft-host interfaces, further exacerbates electrical heterogeneity and facilitates reentry circuits. Together, these mechanisms create a substate that is vulnerable to both focal and reentrant arrhythmias following cell transplantation (Guragain et al., 2024a, 2025; Romagnuolo et al., 2019). Thus, it is essential to analyze abnormal electrical waves to mechanistically elucidate these arrhythmias.

## Experimental techniques to induce and track cardiac electrical waves

Cardiac mapping and optogenetic tools enable investigation of cardiac electrophysiology across multiple spatial and temporal scales. These approaches are used to study fundamental mechanisms of excitation and arrhythmogenesis both *in vitro* and *in vivo*, and to evaluate the integration and safety of cell-based therapies in preclinical transplantation models. *In vitro* systems allow controlled analysis of cellular properties and graft-specific behaviors, whereas *in vivo* and *ex vivo* models provide physiological context to assess graft-host coupling, conduction and arrhythmia susceptibility (Guragain et al., 2024a,b). Together, these techniques enable precise characterization of electrical activity and mechanistic insight into graft-associated arrhythmias following transplantation (Guragain et al., 2025).

### Electrical mapping

Electrical cardiac mapping is widely used clinically to pinpoint sites for arrhythmia ablation procedures (Friedman, 2002; Hertel et al., 2023) and is also used in preclinical research [e.g. in pigs (Marchiano et al., 2023) and non-human primates (Liu et al., 2018)]. Electrode arrays to record electrical signals in the heart can be attached via needles, epicardial patches, basket catheters, endocardial balloons or multielectrode catheters (Malkin et al., 2005). Catheter electrodes can be passed through blood vessels to access the interior of the heart for endocardial mapping. In unipolar mapping, a reference electrode is placed at a remote location (often the body surface), and the recording electrodes are in contact with the heart; thus, the output signals are the extracellular potentials at the recording sites in the myocardium. In bipolar mapping, potentials are recorded between closely spaced pairs of electrodes in contact with myocardium. Bipolar signals are more sensitive to activation wavefronts than unipolar signals, but require more electrodes and lose sensitivity when the wavefront is parallel to the interelectrode axis. Electrical mapping records potential in the extracellular space, which is driven by electrical events in the adjacent myocytes, but is different from the Vm. Vm is directly associated with the membrane biophysics underlying the AP and is generally of more interest. Electrical mapping signals are also susceptible to artifact from electrical stimuli.

### Optical mapping

Optical cardiac mapping is a preclinical investigative technique that can be used to directly monitor Vm, intracellular CaTs or both simultaneously. It has greater spatial resolution than electrical mapping and has been applied to many *in vivo* and *in vitro* preparations including *ex vivo* whole hearts, *in situ* whole hearts, isolated atria, wedge preparations, cardiac slices and cellular monolayers (O'Shea et al., 2020). In any experimental setting, the basic apparatus consists of (1) a myocardial preparation loaded with a fluorescent indicator, often for Vm; (2) an optical setup for delivering excitation light; and (3) a photodetector for collecting the emitted fluorescence (O'Shea et al., 2020). Excitation and emission filters are used to reduce background fluorescence and maximize the signal-to-noise ratio. Commonly used high-speed photodetectors include charged-coupled device (CCD) and complementary metal oxide semiconductor (CMOS) cameras (Efimov et al., 2004). Although the technique has largely been limited to *ex vivo* studies, the development of new fluorescent indicators, imaging systems and computational methods has recently enabled experiments to be performed in living animals, such as pigs (O'Shea et al., 2020; Zhang et al., 2023a).

Optical mapping usually requires the introduction of one or more fluorescent indicators. They are traditionally organic dyes; but, more recently, genetically encoded indicators have been introduced (O'Shea et al., 2020). The most common voltage-sensitive indicators are organic styryl dyes, such as Di-4-ANEPPS, Di-4-ANEQ(F)PTEA, Di-8-ANEPPS and RH-237, which become highly fluorescent upon binding to membranes. A hydrocarbon chain anchors the dye molecule to the cell membrane with the fluorophore oriented perpendicular to the membrane surface and parallel to the membrane potential gradient (Loew, 2015). When the fluorophore is excited by a photon of light, an electric charge travels across the molecule. The energy required to move the charge across the molecule depends on Vm; thus, the dye's excitation and emission spectra shift in proportion to Vm. The most commonly used Ca^2+^-sensitive indicators are the organic dyes Fura-2, Rhod-2, Fluo-4 and Cal-520 (Jaimes et al., 2016; Tada et al., 2014). Combinations of voltage- and Ca^2+^-sensitive dyes can be used to monitor Vm and CaTs simultaneously, which is particularly useful for studies of excitation-contraction coupling (Jaimes et al., 2016). For example, the voltage- and Ca^2+^-sensitive dyes RH-237 and Rhod-2, respectively, are excited by the same wavelength of light but have different emission wavelengths. Simultaneous voltage and Ca^2+^ data can be collected with a single illumination source combined with a dichroic mirror to separate emission light by wavelength and direct it to two photodectectors (Choi and Salama, 2000; Herron et al., 2012). Some investigators have added the capacity to image cellular metabolism to optical mapping studies by taking advantage of the intrinsic fluorescence of nicotinamide adenine dinucleotide plus hydrogen (NADH) and imaging its dynamics together with Vm and CaT (George et al., 2022).

The duration of the experimental recording period for both voltage- and Ca^2+^-sensitive dyes is constrained by photobleaching, phototoxicity, and dye internalization or washout (Matiukas et al., 2007; O'Shea et al., 2020). These limitations can be overcome by using gene-editing techniques to fuse Vm- or CaT-sensitive proteins to fluorescent proteins (O'Shea et al., 2019, 2020). Genetic indicators are typically delivered via viral transduction, including lentivirus and adeno-associated virus (AAV) (Grodem et al., 2023; Kaestner et al., 2014; Oh et al., 2019). Furthermore, cell-type specificity can be achieved via the inclusion of a cell-specific promoter (Kaestner et al., 2014; Skopenkova et al., 2021). For example, the dual-function CaT/Vm indicator (CaViar) can be targeted to cardiomyocytes via the myosin light-chain kinase 7 (MHCK7) promoter. This has allowed researchers to map Vm and CaTs in dechorionated zebrafish embryos and adults to study heart development *in vivo* and the effects of drugs on heart function (Hou et al., 2014). Genetic Ca^2+^ indicators are currently better developed than Vm indicators. Genetic voltage indicators (e.g. VSFP2.3 and ArcLight) (Entcheva and Kay, 2021; Oh et al., 2019) can interfere with the native electrophysiology and are less effective for monitoring rapid kinetics.

Genetic Ca^2+^ indicators generally consist of three components: (1) a circularly permuted fluorescent protein; (2) calmodulin, which has four Ca^2+^-binding sites that regulate the conformational and fluorescence properties of the fused dye protein; and (3) a calmodulin-binding domain (e.g. M13 and RS20) (Oh et al., 2019). GCaMP1 (Nakai et al., 2001) contains the fluorescent protein GFP and is the most widely used genetic Ca^2+^ indicator in cardiac studies (Entcheva and Kay, 2021), although more recent GFP-based variants (e.g. GCaMP8) (Zhang et al., 2023b), as well as red-shifted genetic Ca^2+^ indicators (e.g. jRCaMP1a, jRCaMP1b) (Dana et al., 2016), have also been developed (Entcheva and Kay, 2021). Notably, some genetic Ca^2+^ indicators outperform their synthetic dye equivalents and are also suitable for long-term *in vivo* experiments, for example, to monitor conduction abnormalities and track the integration of transplanted cells for cardiac repair in animal models (Chen et al., 2013; Entcheva and Kay, 2021). They are also suitable for studies of other cell types and organelles (nuclei, mitochondria or the sarcoplasmic reticulum) (Entcheva and Kay, 2021).

### Electrical and optical stimulation

Experimental stimulation of cardiac tissue is necessary to precisely control the timing and location of electrical activation, enabling systematic assessment of conduction properties, excitability and arrhythmia susceptibility. Cardiac tissue is traditionally stimulated electrically, either with field stimulation, which applies a relatively uniform electric field to a large region of tissue (Tandon et al., 2009), or with focal stimulation, which delivers the electrical stimulus to a specific, localized area (Kang et al., 2016). The former is often intended to activate an entire preparation at once, while the latter typically launches an activation wave from the stimulus site.

Optogenetics is an alternative technology in which light-sensitive proteins (opsins) are expressed in targeted cells (Alekseev et al., 2026; Baines et al., 2024; Entcheva and Kay, 2021; Gracheva et al., 2024). The method enables cells to be stimulated by light with precise temporal and spatial control. The technique was initially developed for neuroscience applications but has since been adapted for use in ophthalmology and cardiovascular studies (Emiliani et al., 2022). Optogenetic stimulation can be combined with optical mapping for all-optical electrophysiological studies. Studies in *Caenorhabditis elegans* (Akerboom et al., 2013) and cultured rat neurons (Hochbaum et al., 2014) demonstrated the feasibility of combining opsin ‘actuators’ with genetic voltage and Ca^2+^ indicators, thereby enabling membrane depolarization and CaT activity to be both induced and monitored optically. Furthermore, optogenetics enables more precise control of different cell populations than electrical stimulation. Experiments in mouse brain tissue (Klapoetke et al., 2014) showed that when two genetic actuators (the channelrhodopsins Chronos and Chrimson) were expressed in different sets of neurons within the same cortical microcircuit layer, each actuator could be driven independently by illumination with blue (Chronos) or red (Chrimson) light with no overlap between the blue- and red-light-sensitive cell populations.

Cardiac optogenetics emerged in 2010 when channelrhodopsin-2 (ChR2) (Nagel et al., 2003), an opsin that allows Na^+^ and hydrogen (H^+^) ions to enter a cell in response to blue light, was used as an optogenetic actuator to stimulate zebrafish cardiomyocytes (Arrenberg et al., 2010; Bruegmann et al., 2010). Subsequent experiments demonstrated that when non-excitable human embryonic kidney 293 (HEK293) cells were engineered to express ChR2 and CX43, the cells formed gap junctions with adult canine cardiomyocytes and, when illuminated with blue light, triggered both cardiomyocyte APs and contractile activity (Jia et al., 2011). Since then, optogenetics has been used to interrogate electrophysiology in diverse experimental platforms including *in vitro* systems, such as isolated cardiomyocytes and engineered cardiac tissues, as well as *ex vivo* hearts and *in vivo* small-animal models (e.g. mice, rats and zebrafish). Such studies have combined optogenetic actuators with synthetic dyes (Dong et al., 2019; Klimas et al., 2016; Marchal et al., 2023) or genetic voltage and Ca^2+^ indicators (Dempsey et al., 2016; Fink et al., 2026; Xiao et al., 2025) for optical Vm mapping (Gruber et al., 2022; Hochbaum et al., 2014; Li et al., 2017), CaT mapping (Afshar Saber et al., 2018; Akerboom et al., 2013; Jia et al., 2011) or both (Bruegmann et al., 2010; Dempsey et al., 2016).

Optogenetics can be combined with optical mapping to enable all-optical stimulation and sensing. For example, our laboratory recently developed a method that combines optogenetic stimulation with simultaneous Vm/CaT mapping (Guragain et al., 2024b). It utilizes the optogenetic actuator CheRiff (blue-shifted channelrhodopsin variant) and the genetically encoded calcium indicator janelia red calcium indicator protein 1b (jRCaMP1b). Vm is detected by staining with the organic dye N-(4-sulfobutyl)-4-(6-(4-(dibutylamino)phenyl)hexatrienyl)pyridinium (RH237). Vm and CaT can be recorded simultaneously without crosstalk. This concept was also used for high-throughput drug screening with the all-Optical Dynamic Cardiac Electrophysiology (OptoDyCE) system (Klimas et al., 2016). In a proof-of-concept study, ChR2-expressing cardiomyocytes derived from neonatal rat ventricular myocytes or pluripotent stem cells, together with synthetic voltage- and Ca^2+^-sensitive dyes, were used in a scalable 96-well platform to detect Vm and CaT changes following treatment with varying doses of the antiarrhythmic agent nifedipine. In another study, ventricular arrhythmias evoked by electrical stimulation were effectively terminated via epicardial illumination in transgenic mice with ChR2-expressing cardiomyocytes and in wild-type mice that had been treated with AAVs coding for ChR2 (Bruegmann et al., 2016). Thus, optogenetic technology may one day have direct clinical applications (Boyle et al., 2018; Bruegmann et al., 2022) including, perhaps, as a feasible alternative to implanted electrical defibrillators for patients with heart disease who are at risk of fatal arrhythmia (Guragain et al., 2024b).

### Summary

Graft-associated arrhythmias can be mechanistically elucidated using a variety of high-resolution electrical and optical mapping techniques in combination with optogenetic tools, which enable precise spatiotemporal characterization and targeted modulation of graft-host electrical activity to identify sources of abnormal conduction and ectopic firing following cell transplantation. A summary of the key experimental techniques used to induce and track cardiac electrical waves is provided in Table 1.

**Table 1.** Summary of key experimental techniques to induce and track cardiac electrical waves

Technique	Main uses	Benefits	Limitations
Electrical mapping (e.g. multi-electrode arrays, intracardiac electrodes) (Friedman, 2002; Hertel et al., 2023)	Record extracellular field potentials; map conduction velocity and activation patterns in tissue or whole heart	High temporal resolution; direct measurement of electrical activity; clinically translatable	Limited spatial resolution (depends on electrode density); poor depth resolution; tissue contact required; signal distortion from electrode placement
Optical mapping (Vm- or CaT-sensitive dyes or genetic indicators) (Efimov et al., 2004; O'Shea et al., 2020)	Visualize spatiotemporal propagation of action potentials and CaTs across tissue	High spatial resolution; enables whole-heart mapping; captures wave dynamics (reentry, automaticity, triggered activity)	Requires dyes (potential phototoxicity) or genetic encoding; motion artifacts; often requires *ex vivo* preparations with suppressed cardiac motion
Electrical stimulation (pacing electrodes) (Kang et al., 2016; Tandon et al., 2009)	Induce action potentials; control heart rhythm; test conduction properties and arrhythmia susceptibility	Simple and well established; precise timing control; clinically relevant	Limited spatial specificity; can introduce stimulation artifacts
Optical stimulation (optogenetics) (Alekseev et al., 2026; Entcheva and Kay, 2021; Guragain et al., 2024b; Nagel et al., 2003)	Use light-sensitive ion channels (e.g. channelrhodopsin) to control excitation and wave propagation	High spatial and temporal resolution; non-contact stimulation; cell-type specificity possible	Requires genetic modification; limited light penetration; specialized equipment needed
Combined optical mapping+stimulation (all-optical electrophysiology) (Klimas et al., 2016)	Simultaneous stimulation and recording of cardiac activity using light-based tools	Enables cell-specific control, high spatiotemporal resolution; minimal stimulation artifacts	Technically complex; phototoxicity risk; requires careful spectral separation and system calibration

CaT, calcium transient; Vm, membrane potential.

## Transplantation of stem cell-derived cardiomyocytes

The cardiomyocyte sources used in these experimental approaches have evolved substantially over time. Early transplantation studies relied on primary or tumor-derived cardiomyocytes, which were limited by low proliferative capacity and availability (Koh et al., 1993). Subsequent advances in stem cell biology, including the differentiation of cardiomyocytes from embryonic stem cells and induced pluripotent cells, have enabled scalable cell production. As differentiation protocols have improved, human induced pluripotent stem cell-derived cardiomyocytes (hiPSC-CMs) have become the predominant cell type for *in vitro* studies and preclinical cardiac transplantation models (Zhang et al., 2024).

The first report (Koh et al., 1993) of cardiomyocyte transplantation was published in 1993, when mouse cardiomyocytes were implanted in the hearts of syngeneic mice and viable grafts remained detectable up to 4 months later. However, the nearly nonexistent proliferative capacity that prevents endogenous cardiomyocytes from repopulating the myocardial scar also prevents primary cardiomyocytes from being expanded in culture. This severely limits their availability for experiments and may partially explain why this pioneering investigation was performed with a tumor-cell line (AT-1 cardiomyocytes) that was capable of proliferation. Cardiomyocytes remained scarce until the development of methods for differentiating them from stem cells, initially embryonic stem cells (ESCs) (He et al., 2003; Mummery et al., 2003) and then induced-pluripotent stem cells (iPSCs) (Takahashi et al., 2007; Takahashi and Yamanaka, 2006; Yu et al., 2007). Cardiomyocytes differentiated from human ESCs (hESC-CMs) were first developed and shown to form gap junctions with fetal cardiomyocytes in 2002 (Mummery et al., 2003). HESC-CM APs were characterized into three distinct types – nodal, atrial and ventricular – in 2003 (He et al., 2003). iPSCs were reprogrammed from mouse fibroblasts (miPSCs) in 2006 (Takahashi and Yamanaka, 2006) and from human fibroblasts (hiPSCs) in 2007 (Takahashi et al., 2007; Yu et al., 2007). As protocols for differentiating hiPSCs into cardiomyocytes (hiPSC-CMs) and other cell types have progressively improved (Kahn-Krell et al., 2021; Masumoto et al., 2014; Ren et al., 2011; Sharma et al., 2015; Zahanich et al., 2011; Zhao et al., 2019), hiPSC-CMs have emerged as the most commonly used cardiomyocytes for implantation studies or as components of tissue-engineered cardiac-muscle patches (Zhang et al., 2024).

Preclinical investigations of transplanted hiPSC-derived cells (or engineered myocardial tissues that have been constructed with hiPSC-derived cells) are performed in either immunocompromised animals or with concomitant immunosuppressive therapy. Nevertheless, the proportion of cells that are retained and survive at the site of administration (i.e. the engraftment rate) is unacceptably low. Notably, when cardiomyocytes were differentiated from hiPSCs that overexpressed the cell-cycle regulatory molecule cyclin D2 (CCND2) and implanted into the infarcted hearts of immunodeficient mice, the small initial population of engrafted hiPSC-CMs grew to repopulate more than 50% of the myocardial scar over the ensuing 6 months (Fan et al., 2019; Zhu et al., 2018). However, even with such strategies, the engraftment rate is likely to be much lower in immunocompetent animals, and some evidence suggests that even autologous iPSC-derived cells may be immunogenic. For instance, miPSCs reprogrammed from C57BL/6 mouse fibroblasts induced an immune response when delivered to syngeneic C57BL/6 recipients (Zhao et al., 2011). Furthermore, because iPSCs can proliferate to all cell lineages, a small population of cells within an hiPSC-CM population that fail to completely differentiate could (at least in principle) be tumorigenic (Heslop et al., 2015; Lee et al., 2013; Tang et al., 2018; Zhang et al., 2011). Thus, although tumors have never, to our knowledge, been observed in animals after treatment with hiPSC-CMs, the potential risk cannot be entirely disregarded (Heslop et al., 2015; Kawaguchi et al., 2021; Lee et al., 2013; Tang et al., 2018; Zhang et al., 2011).

hiPSC-CMs also tend to be developmentally immature and more closely resemble fetal cardiomyocytes than the cardiomyocytes of developmentally mature mammals (Ahmed et al., 2020; Tan and Ye, 2018; Wu et al., 2021), which could lead to electrophysiological complications. For example, mature ventricular cardiomyocytes do not beat spontaneously, but both fetal cardiomyocytes and hiPSC-CMs retain some automaticity (Ahmed et al., 2020; Tan and Ye, 2018; Wu et al., 2021), which could increase the risk of ectopic heartbeats. The APs of hiPSC-CMs also differ substantially from those of mature cardiomyocytes, with slower conduction velocity (10-20 versus 60 cm/s, respectively), reduced upstroke velocity (maximum rate of rise of the AP during depolarization phase, 50 versus 250 V/s, respectively), and higher membrane potentials (–60 versus −90 mV, respectively) reflecting their more immature electrophysiological phenotype (Jiang et al., 2018). Thus, strategies to promote hiPSC-CM maturation continue to be actively explored and are likely to be crucial for the therapeutic translation of hiPSC-CMs, as well as their utility as *in vitro* models of cardiac disease. hiPSC-CM maturation can be promoted using a combination of biophysical, biochemical and metabolic cues that mimic postnatal cardiac development (Hong et al., 2023). Common approaches include electrical and mechanical stimulation, long-term culture, three-dimensional (3D) engineered tissue systems, and metabolic maturation strategies that shift energy substrate utilization from glucose to fatty acids, thereby promoting more adult-like electrophysiological and contractile properties (Kahn-Krell et al., 2021; Yang et al., 2023).

## Electrophysiological observations from preclinical studies of cardiomyocyte transplantation

Although early studies found no increased risk of arrhythmia in small animals that had been treated with human cardiomyocytes (Chong et al., 2014), engraftment-related arrhythmias have been reported in pigs (Kawaguchi et al., 2021; Selvakumar et al., 2024) and non-human primates, which are more clinically relevant models of human disease (Chong et al., 2014; Kobayashi et al., 2024; Liu et al., 2018; Shiba et al., 2012, 2016). The arrhythmias had the highest incidence 1-2 weeks after transplantation, followed by a gradual decline in arrhythmia burden over time (Chong et al., 2014; Romagnuolo et al., 2019; Shiba et al., 2016). Although these events can often be transiently suppressed with standard antiarrhythmic drugs, such as ivabradine and amiodarone, they are not completely eliminated, and it is crucial to fully eliminate such early arrhythmic events (Liu et al., 2018; Selvakumar et al., 2024). Some reports have also shown an increase in arrhythmia that is associated with graft size (Kobayashi et al., 2024; Liu et al., 2018). The spatial distribution of transplanted cells may influence arrhythmia risk, as clustered or patchy engraftment may create regions of discontinuous conduction (Guragain et al., 2025). Furthermore, the host myocardial substrate may play a key role, with preexisting scar heterogeneity, fibrosis and altered conduction properties providing a vulnerable environment that could interact with graft-induced electrical instability and facilitate reentry (Guragain et al., 2025; Kobayashi et al., 2024; Liu et al., 2018). Thus, arrhythmogenic complications may be the chief safety concern associated with cardiomyocyte-based cardiac therapies. The earliest clinical trials of autologous cell-based cardiac therapies investigated the implantation of bone-marrow cells and skeletal myocytes after MI (Murry et al., 2005), but neither cell type acquired a cardiac-cell phenotype or coupled electrically with the native myocardium (Leobon et al., 2003; Murry et al., 2004; Nygren et al., 2004; Reinecke et al., 2002). Poor coupling between transplanted and host cells can electrically isolate clusters of cells from the native cardiac tissue, and functional differences between electrical properties of transplanted and native tissue can lead to mismatched wave conduction characteristics. Either situation can facilitate reentrant arrhythmias (Almeida et al., 2015; Paci et al., 2020; Yanamandala et al., 2017; Zimmermann, 2020), and, indeed, ventricular tachycardia (VT) was reported in a number of patients who were treated with skeletal myocytes (Hagege et al., 2006; Smits et al., 2003). Subsequent preclinical experiments demonstrated that the incidence of VT induced by programmed electrical stimulation was reduced in infarcted mouse hearts injected with primary embryonic cardiomyocytes or with skeletal myocytes genetically engineered to express CX43 (Roell et al., 2007). The researchers of this study did not evaluate contractile performance, but the results demonstrated that transplanted cardiomyocytes can improve the heart's electrophysiological activity, and that CX43 expression is crucial for intercellular coupling.

In a mouse MI model, VT incidence was greater when the animals were treated with mouse embryonic stem cell-derived cardiomyocytes (mESC-CMs) than in either the absence of treatment administration or in a group that received undifferentiated ESCs (Liao et al., 2010). The authors attributed the VT increase to an immature cardiomyocyte phenotype and later showed that it was attenuated by K^+^ channel overexpression, which would be characteristic of a more mature phenotype (Liao et al., 2013). In a study from our laboratory that implanted relatively immature hiPSC-CMs into swine hearts, the engrafted cells exhibited reduced CX43 expression, weaker gap junction formation and slower conduction velocity compared with the host heart. All of these factors are potentially proarrhythmic (Guragain et al., 2025).

Notably, the results from studies using mESC-CMs conflict with studies using human stem cell-derived cardiomyocytes. Neither hESC-CMs nor hiPSC-CMs have been associated with elevated VT risk in small-animal models, likely owing to smaller heart size and higher heart rate in smaller species such as mice and rats, which have smaller spatial scale for sustaining reentry circuits compared with larger species such as pigs. Additionally, hESC-CM transplantation reduced VT incidence in guinea pigs (Shiba et al., 2012). Furthermore, some preclinical investigations (Hodgkinson et al., 2016; Li et al., 2021; Ratajczak et al., 2012; Tan et al., 2021; Tao et al., 2021) have also found evidence of improvement in myocardial contractile activity after treatment with hESC-CMs (Caspi et al., 2007; Shiba et al., 2012; Tachibana et al., 2017), hiPSC-CMs (Funakoshi et al., 2016; Tachibana et al., 2017), or engineered tissues composed of hESC-CMs/hiPSC-CMs and other cell types (Pecha et al., 2019; Riegler et al., 2015; Weinberger et al., 2016) in small-animal models including mice, rats and guinea pigs. However, engraftment rates were typically low, suggesting that the observed improvements were driven primarily by paracrine effects rather than direct electromechanical integration of transplanted cells. The secreted factors from transplanted cells can enhance endogenous cardiomyocyte survival and proliferation, reduce inflammation and fibrosis, and promote vascular growth, thereby improving myocardial contractility by preserving viable tissue and enhancing perfusion (Tachibana et al., 2017). However, improved graft survival and stable electrical coupling may be required to achieve sustained functional recovery beyond these transient, paracrine-mediated benefits (Guragain et al., 2025; Liu et al., 2018; Tachibana et al., 2017).

In large-animal models (both pigs and monkeys), hESC-CM/hiPSC-CM transplantation has been consistently associated with improvements in both cardiac function and infarct size, but increases in arrhythmia risk have also been reported, particularly during the first 2 weeks after cell administration (Chong et al., 2014; Romagnuolo et al., 2019; Shiba et al., 2016). In addition to the risk of reentrant arrhythmias, if engrafted cells possess some potential for spontaneous electrical activity, they could function as a second source of AP wave generation that interferes with the waves produced by the heart's endogenous pacemaker cells (Almeida et al., 2015; Liao et al., 2010; Liu et al., 2018; Marchiano et al., 2023; Romagnuolo et al., 2019). As mentioned earlier, the potential for automaticity may be higher for cardiomyocytes that are relatively immature or have been differentiated from stem cells (Zahanich et al., 2011). In hESC-CM-treated monkeys, electrical mapping localized the origin of ventricular arrhythmias to a focal point source acting as an ectopic pacemaker (Liu et al., 2018). To address this, investigators generated a line of hiPSC-CMs that contracted only in response to external electrical stimulation, but not spontaneously. This was achieved through systematic knockout of genes that promote depolarization: hyperpolarization-activated cyclic nucleotide-gated cation channel 4 (*HCN4*), calcium voltage-gated channel subunit alpha1H (*CACNA1H*) and solute carrier family 8 member A1 (*SLC8A1*), combined with the overexpression of potassium voltage-gated channel subfamily J member 2 (*KCNJ2*), which promotes hyperpolarization. These genetically modified hiPSC-CMs were capable of electromechanical coupling with host cardiomyocytes without inducing sustained arrhythmias (Marchiano et al., 2023). Furthermore, studies in pigs (Selvakumar et al., 2024) identified a unique pattern of surface-marker expression that could distinguish between arrhythmogenic [signal regulatory protein α (SIRPA)^+^CD90^−^CD200^+^] and non-arrhythmogenic (SIRPA^+^CD90^−^CD200^–^) hiPSC-CMs, which suggests that the risk of hiPSC-CM-associated arrhythmia could be substantially reduced by screening the cells for specific patterns of marker expression before administration. hiPSC-CMs also appear to be less arrhythmogenic when co-administered with other cell types, such as endothelial cells (Cheng et al., 2023) or both endothelial and smooth-muscle cells (Ye et al., 2014), because these supporting cells may help create a more physiologically relevant and stable microenvironment that enhances vascularization and improves oxygen and nutrient delivery. They may also secrete paracrine factors that promote cardiomyocyte maturation and reduce arrhythmogenesis (Karbassi et al., 2020; Tachibana et al., 2017).

To aid the design of anticipated clinical trials, a 2024 investigation in monkeys (Kobayashi et al., 2024) was conducted to compare the efficacy and safety of spheroids containing two different doses of hiPSC-CMs (20 million or 60 million cells). The spheroids were implanted 2 weeks after experimentally induced MI. Both doses significantly improved cardiac function following treatment, with greater and more sustained benefits observed in the higher-dose group. Two of the four high-dose recipients experienced sustained VT (maximum rate of 176 beats/min), but the risk was considered acceptable because such arrhythmias can be managed by defibrillator implantation, and none of the events occurred more than 2 weeks after transplantation, with the longest lasting less than 50 min. Although the results from more than two decades of research in small- and large-animal models suggest that hiPSC-CMs are likely to be suitable for early-stage clinical trials, none of these studies were able to precisely examine the characteristics of the electrical propagation passing through the interface between the host myocardium and grafts.

This lack of data is likely due to inherent limitations in available experimental techniques, such as insufficient high-resolution optical mapping and the inability to record signals from deeper regions at the host-graft interface. Traditional whole-heart electrical and optical mapping techniques that have been used to study graft-associated arrhythmia offer their highest resolution when mapping the heart's endocardial or epicardial surfaces. However, they have limited efficacy in deciphering the fine details of graft-host coupling and graft-associated arrhythmogenesis, particularly when the grafts are implanted deep within the ventricular wall. Our laboratory recently addressed this limitation using an organotypic tissue slice preparation. Organotypic tissue slices are thin slices of myocardium, typically harvested parallel to the epicardium from relevant animal models. Slices maintain the native architecture of the extracellular matrix (Kang et al., 2016; Meki et al., 2021), and, because they provide a multicellular environment, any phenotypic properties that may be unique to a specific subpopulation of cardiomyocytes are likely to be preserved (Ou et al., 2019; Pitoulis et al., 2020; Wang et al., 2015; Watson et al., 2019). Therefore, tissue slices are a powerful system for numerous applications, including *ex vivo* studies of cardiac electrophysiology (Kang et al., 2016; Meki et al., 2021; Perbellini and Thum, 2020; Watson et al., 2020).

**Fig. 2.**
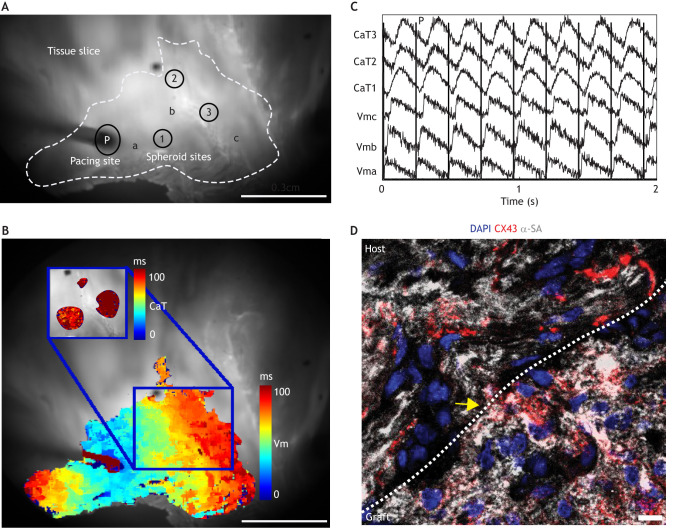
**Electrical coupling between implanted tissue graft and the host myocardium.** (A) A representative fluorescence image of a tissue slice with the mapped region in B outlined by a dashed line. In C, engrafted spheroid CaT traces CaT1, CaT2, and CaT3 were collected from sites 1, 2 and 3, respectively; Vm traces Vma, Vmb, and Vmc were collected from sites a, b and c, respectively. Remote pacing was delivered at site P. Scale bar: 0.3 cm. (B) CaT and Vm activation maps in response to remote 1 Hz remote pacing delivered at site P. A wave propagates left and right from the pacing site. Spheroid sites activate after being reached by the wave (inset). Scale bar: 0.3 cm. (C) CaT and Vm traces for sites 1 to 3 and a to c during remote pacing at 240 beats/min (4 Hz). Note the staggered activation times, which indicates propagation, and phase lock between CaT and Vm signals, which indicates coupling. (D) A cryosection stained with DAPI and antibodies for CX43 (red, which indicates structural integration) and α-SA (cyan, which indicates both human and pig cardiomyocytes). The demarcation between host and graft (dotted line) is evident, showing CX43 bridging the host and graft (arrow). Scale bar: 10 μm. α-SA, α-sarcomeric actin; CaT, Ca^2+^ transient; CX43, connexin-43; DAPI, 4′,6-diamidino-2-phenylindole; Vm, membrane potential. Adapted with permission from Guragain et al. (2024a). This image is not published under the terms of the CC BY license of this article. For permission to reuse, please see Guragain et al. (2024a).

In our recent study from a pig model of MI using myocardial tissue slices, we applied high-resolution optical mapping to assess electrical integration of implanted cardiac spheroids differentiated from hiPSCs 1 week after transplantation (Fig. 2A) (Guragain et al., 2024a). By engineering graft tissue to express a fluorescent Ca^2+^ indicator, we were able to distinguish graft activation from host myocardium and directly evaluate conduction across the host-graft interface (Fig. 2B). Our findings demonstrate that engineered tissue can establish electrical coupling with the host and sustain activation at heart rates up to 240 beats/min (Fig. 2C). However, this coupling remained incomplete, as evidenced by sparse CX43 expression, discontinuous conduction bridges (Fig. 2D), and conduction velocities within the graft being threefold to fivefold slower than in host myocardium (graft, 14-20 mm/s; host, 50-70 mm/s) (Guragain et al., 2024a, 2025). These properties create a substrate for conduction heterogeneity and potential reentry, consistent with prior large-animal transplantation studies reporting early graft-associated arrhythmias and incomplete electrical integration (Chong et al., 2014; Kobayashi et al., 2024; Romagnuolo et al., 2019).

Unlike earlier studies, which primarily inferred graft-host coupling from electroanatomical or lower-resolution mapping approaches, our study provided the first high spatial resolution (∼50 μm) imaging of conduction at the host-graft interface (Guragain et al., 2025), enabling direct visualization of electrical coupling and conduction barriers. Although spontaneous automaticity was observed within graft tissue, we did not detect clear evidence of graft-to-host conduction under these conditions. However, independent pacing of graft was not feasible using conventional electrical stimulation. This limitation highlights the need for more selective stimulation approaches, such as optogenetic methods (Guragain et al., 2024b), to definitively assess graft-driven activation of host myocardium. Collectively, these findings support the idea that early grafts of electrically immature and heterogeneously coupled tissue can contribute to arrhythmogenic substrates, while also emphasizing key differences in resolution and mechanistic insight compared to prior large-animal studies. A summary comparison of electrophysiological outcomes after cardiomyocyte transplantation in different animal model systems is illustrated in Table 2.

**Table 2.** Comparison of electrophysiological outcomes after cardiomyocyte transplantation in different animal model systems

Model system	Cell type(s)	Main electrophysiological findings	Arrhythmia risk	Key mechanistic insights	Limitations
Small animals (mice, rats) (Caspi et al., 2007; Funakoshi et al., 2016; Liao et al., 2010, 2013; Tachibana et al., 2017)	hESC-CMs, hiPSC-CMs, mESC-CMs	No VT	Low overall	Immature phenotype drives automaticity; maturation reduces VT; small heart size limits reentry circuits	Limited clinical relevance; high heart rate and small heart size prevent reentry circuits
Guinea pigs (Shiba et al., 2012)	hESC-CMs	Reduced VT incidence; improved electrical stability	Low	Better scale for reentry than mice; improved coupling reduces arrhythmia	Still smaller heart than human; partial translation
Large animals (pigs) (Guragain et al., 2024a; Guragain et al., 2025; Marchiano et al., 2023; Selvakumar et al., 2024)	hiPSC-CMs	Improved cardiac function; frequent early arrhythmias (1-2 weeks); conduction heterogeneity	Moderate to high (early phase)	Arrhythmias linked to graft size, spatial distribution and incomplete coupling (low CX43, slow conduction); arrhythmogenic cell subpopulations	High cost; low cell retention; variability in graft integration
Non-human primates (monkeys) (Chong et al., 2014; Kobayashi et al., 2024; Liu et al., 2018; Shiba et al., 2016)	hESC-CMs, hiPSC-CMs	Functional improvement; focal VT from ectopic pacemakers; dose-dependent arrhythmia	Moderate to high (transient)	Automaticity of immature grafts; focal ectopic sources; genetic modification in cells reduces arrhythmia	Ethical and logistic constraints; high cost; small sample sizes

hESC-CM, human embryonic stem cell-derived cardiomyocyte; hiPSC-CM, human induced pluripotent stem cell-derived cardiomyocyte; mESC-CM, mouse embryonic stem cell-derived cardiomyocyte; VT, ventricular tachycardia.

**Fig. 3.**
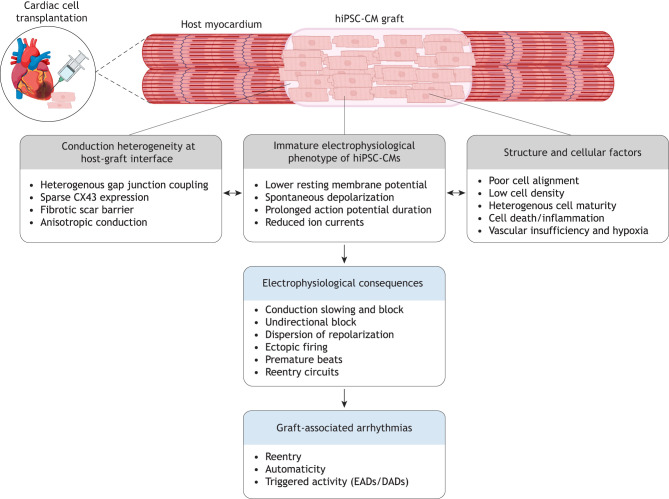
**Summary of major mechanisms of graft-associated arrhythmias and their interrelationships.** Graft-associated arrhythmias arise from multiple interacting mechanisms including conduction heterogeneity at the host-graft interface, the immature electrophysiological phenotype of transplanted hiPSC-CMs, and adverse structural and cellular factors. Together, these conditions disrupt electrical propagation and promote reentry, abnormal automaticity and triggered activity (EADs/DADs). CX43, connexin-43; DAD, delayed afterdepolarization; EAD, early afterdepolarization; hiPSC-CM, human induced pluripotent stem cell-derived cardiomyocytes. Created in BioRender by Guragain, B. (2026). https://BioRender.com/xiw7nob. This figure was sublicensed under CC-BY 4.0 terms.

Preclinical studies of cardiomyocyte transplantation reveal that graft-associated arrhythmias arise from multiple interacting mechanisms. Conduction heterogeneity at the host-graft interface is driven by uneven gap junction coupling, reduced CX-43 expression, fibrotic barriers, and anisotropic conduction that disrupts electrical propagation (Guragain et al., 2025). Transplanted hiPSC-CMs further contribute owing to their immature electrophysiological phenotype, including depolarized resting membrane potential, spontaneous activity, prolonged APs and reduced ion currents (Ahmed et al., 2020; Wu et al., 2021). Structural factors – such as poor alignment, low cell density, heterogeneous maturity, inflammation and hypoxia – exacerbate this instability. Together, these conditions promote reentry, abnormal automaticity and triggered activity (EADs/DADs), leading to graft-associated arrhythmias (Fig. 3) (Guragain et al., 2025).

The experimental evidence across model systems highlights important differences in the mechanisms and severity of graft-associated arrhythmias. Small-animal models (e.g. mice and rats) are highly useful for mechanistic insight but often overestimate arrhythmia burden due to faster intrinsic heart rate, shorter APs, species-specific conduction properties and limited heart size (Shiba et al., 2012). In contrast, large-animal models (e.g. pigs and non-human primates) more closely resemble human heart size, conduction velocity and autonomic regulation, providing a more clinically relevant assessment of graft-host electrical integration and arrhythmia risk (Guragain et al., 2024a, 2025; Liu et al., 2018). Consequently, findings from small-animal studies should be interpreted cautiously and validated in large-animal models to better predict human translational outcomes.

## Clinical trials of hiPSC-CM transplantation

The first clinical trial of hiPSC-CM transplantation in patients with heart failure is currently being conducted in Japan (Cyranoski, 2018; Eschenhagen et al., 2022). A sheet composed of 100 million allogeneic hiPSC-CMs will be injected epicardially, with concomitant immunosuppressive therapy administered for 4 weeks after cell delivery. The results from studies in pigs suggest that the transplanted cells will improve cardiac function via the release of paracrine factors, rather than by integrating with the native myocardium. Göttingen University (Germany) has initiated an investigation of an engineered heart-tissue construct composed of hiPSC-CMs and stromal cells in patients with end-stage heart failure (ClinicalTrials.gov ID NCT04396899; Carvalho, 2023; Eschenhagen et al., 2022; Jebran et al., 2025; Mallapaty, 2020), aimed primarily at assessing safety, graft retention and functional integration. The biotech firm Heartseed and the Danish pharmaceutical company Novo Nordisk have initiated the phase 1/2 LAPiS trial: a dose-escalation study of hiPSC-CM spheroid administration in up to ten patients with severe heart failure due to ischemic heart disease (ClinicalTrials.gov ID NCT04945018; Carvalho, 2023; Zhang et al., 2024). The trial will be conducted in nine clinical centers in Japan, and the treatment – a highly purified preparation of ventricular hiPSC-CM spheroids – will be delivered via intramyocardial injection with a specialized dome-shaped needle during open heart surgery. The trial strategy was based on previous work showing that transplanted hiPSC-CM spheroids improved cardiac function in pigs and rodents (Kawaguchi et al., 2021) and are more readily engrafted than suspensions of single cells (Carvalho, 2023). Help Therapeutics in China is also conducting a trial of hiPSC-CM administration in patients with heart disease (ClinicalTrials.gov ID NCT04982081; Carvalho, 2023; Mallapaty, 2020), and the approved enrollment was recently increased to 20 patients. Further, Stanford University (USA) has started an early-phase hESC-CM transplantation trial for patients with left-ventricular dysfunction to identify the maximum tolerated dose for safety and feasibility (ClinicalTrials.gov ID NCT05068674). A summary of key clinical trials of hiPSC-CM-based therapies, including trial phase and preliminary findings, is provided in Table 3.

**Table 3.** Clinical trials of hiPSC-CM transplantation for cardiac repair

Trial and start date	Location	Phase	Cell type and delivery method	Patient population	Preliminary findings
Osaka University (2018) (Cyranoski, 2018; Eschenhagen et al., 2022)	Japan	Phase I (early feasibility)	Allogenic hiPSC-CM sheet (∼100 million cells), epicardial implantation	Ischemic heart failure	First-in-human trial; primarily safety focused; early reports suggest feasibility and no major adverse events; benefits likely by paracrine effects
Gottingen University (2020) (ClinicalTrials.gov ID NCT04396899; Carvalho, 2023; Eschenhagen et al., 2022; Mallapaty, 2020)	Germany	Phase I/ II	Engineered heart tissue (hiPSC-CMs+stromal cells), surgical implantation	End-stage heart failure	First-in-human study of tissue-engineered construct; focuses on safety, graft survival and integration; preliminary findings reported
LAPiS-Heartseed Inc. (2022) (ClinicalTrials.gov ID NCT04945018; Carvalho, 2023; Zhang et al., 2024)	Japan (multi-center)	Phase I/ II	hiPSC-CM spheroids, intramyocardial injection	Heart failure due to ischemic heart disease	Dose-escalation design (∼10 patients); based on preclinical data; aims to assess safety, engraftment and functional improvement
Help Therapeutics (2022) (ClinicalTrials.gov ID NCT04982081)	China	Phase I	Allogenic hiPSC-CMs, intramyocardial/ intracoronary delivery	Heart disease (heart failure)	Enrollment (∼20 patients); early-stage safety and feasibility evaluation
Stanford University (2022) (ClinicalTrials.gov ID NCT05068674)	USA	Phase I	hESC-CMs (50 million to 300 million cells); transendocardial injection	Chronic ischemic left-ventricular dysfunction	Safety and feasibility evaluation; identify maximum tolerated dose

hESC-CM, human embryonic stem cell-derived cardiomyocyte; hiPSC-CM, human induced pluripotent stem cell-derived cardiomyocyte.

Together, these clinical efforts highlight the growing translational momentum in the field, while also underscoring the continued need for rigorous preclinical research to optimize safety, efficacy and long-term functional integration of engineered cardiac therapies. Importantly, a key unsolved question for clinical translation remains the relative contribution of paracrine-mediated signaling versus direct electromechanical integration of transplanted cells, which is critical for determining whether observed functional improvements reflect durable myocardial regeneration or transient supportive effects.

## Summary

MI is characterized by profound mechanical dysfunction, including ventricular wall thinning, adverse remodeling and impaired contractility, as well as electrophysiological disturbances. Over the past three decades, a variety of strategies have been employed to effect cardiac repair. These include cell transplantation (Sadek and Olson, 2020; Ye et al., 2014; Zhao et al., 2021; Zhu et al., 2018), stimulating native cardiomyocyte proliferation (Pasumarthi et al., 2005; Shapiro et al., 2014; Xiang et al., 2016) and reprogramming fibroblasts into cardiomyocytes (Fu and Srivastava, 2015; Tani et al., 2023; Yamakawa and Ieda, 2021; Zhao et al., 2015). Successful cardiac regeneration will require reestablishment of coordinated electromechanical coupling to support synchronous contraction and overall pump function. Cell transplantation has shown promising therapeutic potential with remuscularization and improved cardiac function (Ye et al., 2014). However, the risk of arrhythmias, particularly evident in large-animal models, remains a major barrier to safe clinical translation (Chong et al., 2014; Romagnuolo et al., 2019; Shiba et al., 2016; Guragain et al., 2025). Computational models predict that the arrhythmia burden associated with MI and hiPSC-CM transplantation peaks approximately 14 days after acute MI (Yu et al., 2022), which is consistent with observations in a number of large-animal studies (Chong et al., 2014; Romagnuolo et al., 2019; Shiba et al., 2016). Fortunately, pigs that experienced treatment-related arrhythmia after hiPSC-CM administration responded well to standard pharmacological therapy (ivabradine and amiodarone) (Selvakumar et al., 2024), which suggests that arrhythmic complications in the growing number of clinical trial participants are likely to be manageable. Defibrillators could also be used to mitigate risk during the early post-transplantation period.

Looking forward, several key challenges must be addressed to enable safe and effective clinical translation. First, improving the structural and functional maturity of hiPSC-CMs remains a critical priority, as their immature, fetal-like phenotype contributes to automaticity and incomplete coupling with host myocardium (Marchiano et al., 2023). Approaches such as electrical and biochemical stimulation (Hong et al., 2023), mechanical loading (Hong et al., 2023) and advanced 3D culture platforms (Hong et al., 2023; Kahn-Krell et al., 2021) have shown promise in promoting maturation and more adult-like ion channel expression profiles (Ahmed et al., 2020; Gao et al., 2023; Wu et al., 2021). Second, achieving robust and stable bidirectional electrical coupling between graft and host tissue is essential to ensure synchronized activation and to minimize conduction heterogeneity that can promote arrhythmias (Guragain et al., 2025). Third, defining effective strategies to mitigate arrhythmia risk in clinically relevant settings including optimized cell delivery methods, dosing strategies, and adjunctive pharmacological or device-based interventions remains an area of active investigation. Emerging state-of-the-art optical mapping techniques will accelerate progress by enabling detailed characterization of AP propagation across graft-host interfaces, providing critical mechanistic insights into electromechanical integration. Together, these advances will help guide the rational design of next-generation cell-based therapies that not only restore myocardial tissue but also reconstitute the complex electromechanical function of the heart, ultimately facilitating the safe and durable clinical translation of hiPSC-CM-based regenerative therapies.
